# Chemoimmunotherapy vs Immunotherapy Monotherapy Receipt in Advanced Non–Small Cell Lung Cancer

**DOI:** 10.1001/jamanetworkopen.2024.59380

**Published:** 2025-02-12

**Authors:** Vinayak S. Ahluwalia, Ravi B. Parikh

**Affiliations:** 1 Perelman School of Medicine, University of Pennsylvania, Philadelphia; 2 Leonard Davis Institute of Health Economics, University of Pennsylvania, Philadelphia; 3 Emory University School of Medicine, Atlanta, Georgia; 4 Emory Winship Cancer Institute, Atlanta, Georgia

## Abstract

This cross-sectional study examines factors that may be associated with the prescription of chemoimmunotherapy vs immunotherapy monotherapy in cases of advanced non–small cell lung cancer using nationwide data from the US.

## Introduction

In driver mutation-negative advanced non–small cell lung cancer (aNSCLC), chemotherapy with anti–programmed cell death 1 (PD1) or its ligand (PD-L1) immune checkpoint inhibitor therapy (ICI) is standard of care for patients with PD-L1 expression (ie, tumor proportion score) of less than 50% (PD-L1 low). When PD-L1 expression is 50% or higher (PD-L1 high), ICI monotherapy and chemoimmunotherapy show similar outcomes in the US Food and Drug Administration pooled analyses.^1,2^ Except for scenarios, such as high tumor burden or advanced age, clinical guidelines do not recommend 1 treatment over another, and it is unclear what factors influence prescribing of each treatment in the clinical setting.^3^

## Methods

This cross-sectional study followed the Strengthening the Reporting of Observational Studies in Epidemiology (STROBE) reporting guideline^4^ and was approved by the University of Pennsylvania institutional review board with a waiver for informed consent because data were deidentified. The study used a nationwide electronic health record–derived database of patients receiving first-line therapy for aNSCLC.^5^ We fit a logistic regression model estimating receipt of ICI monotherapy or chemoimmunotherapy in PD-L1 high patients and reported odds ratios (ORs) for relevant covariates. We plotted the proportion receiving ICI monotherapy vs diagnosis year, stratifying by PD-L1 status. Statistical significance was set at *P* < .05. Stata version 18.0 (StataCorp) was used for statistical analysis. Data were analyzed from January 2018 to May 2024. Additional information can be found in the eMethods in Supplement 1.

## Results

In this study, 5448 patients met the inclusion criteria (median [IQR] age at diagnosis, 70 [63-77] years; 2781 males [51.1%]; 611 Black patients [11.2%]; 4370 White patients [80.2%]), 2690 (49.4%) received ICI monotherapy, and 3119 patients (57.3%) were PD-L1 high. Additionally, 1967 patients (63.1%) and 723 patients (31.0%) received ICI monotherapy in the PD-L1 high and PD-L1 low subgroups, respectively. The proportion of PD-L1 high patients who received chemoimmunotherapy increased from 169 of 637 patients (26.5%) in 2018 to 134 of 337 patients (39.8%) in 2023 (*P* < .001) (Figure).

**Figure.**
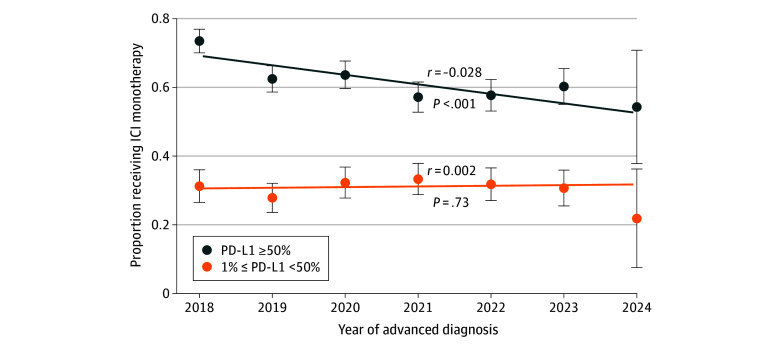
Trends in Immune Checkpoint Inhibitor (ICI) Monotherapy Receipt Over Time Proportion of the patients in the cohort who received ICI monotherapy over time, stratified by programmed cell death–ligand 1 (PD-L1) status. The proportion of patients receiving ICI monotherapy is calculated as (number receiving ICI monotherapy) ÷ (number receiving ICI monotherapy + number receiving chemoimmunotherapy). Bands represent 95% CIs; *r* represents the best-fit linear regression coefficient.

For PD-L1 high patients, factors favoring ICI monotherapy receipt over chemoimmunotherapy included Eastern Cooperative Oncology Group (ECOG) status of 2 or higher (OR, 1.87; 95% CI, 1.32-2.64; *P* < .001), practicing at an academic medical center (OR, 2.66; 95% CI, 1.81-3.92; *P* < .001), age older than 75 years (OR, 2.46; 95% CI, 1.85-3.27, *P* < .001), being female (OR, 1.44; 95% CI, 1.12-1.85; *P* = .004), having brain metastases (OR, 2.14; 95% CI, 1.17-3.91; *P* = .01), and concurrent anti-infective use (OR, 4.04; 95% CI, 2.16-7.56; *P* < .001) (Table). Factors favoring chemoimmunotherapy included membership in the top quintile for socioeconomic status (SES; OR, 0.55; 95% CI, 0.36-0.85; *P* = .007), uninsured status (OR, 0.68; 95% CI, 0.49-0.94; *P* = .02), more recent year of diagnosis (OR, 0.86; 95% CI, 0.80-0.93; *P* < .001), and glucocorticoid use (OR, 0.009; 95% CI, 0.007-0.012; *P* < .001). Kirsten rat sarcoma virus (KRAS) positivity was not associated with treatment receipt.

**Table.** ORs Derived From Logistic Regression Relating Likelihood of Receiving ICI Monotherapy vs Chemoimmunotherapy in Patients With PD-L1 of 50% or Higher

Covariate	OR (95% CI)	*P* value
Socioeconomic status		
1 (Lowest)	1 [Reference]	NA
2	0.86 (0.58-1.29)	.47
3	1.08 (0.72-1.61)	.72
4	0.89 (0.59-1.33)	.56
5 (Highest)	0.55 (0.36-.85)	.007
ECOG		
0	1 [Reference]	NA
1	1.06 (0.79-1.44)	.69
≥2	1.87 (1.32-2.64)	<.001
Tumor histology		
Squamous cell histology	1 [Reference]	NA
Nonsquamous cell histology	0.79 (0.58-1.09)	.15
Ethnicity		
Non-Hispanic or Latino	1 [Reference]	NA
Hispanic or Latino	1.00 (0.46-2.15)	.99
Self-identified race		
Asian	1.05 (0.41–2.70)	.93
Black	1.06 (0.71-1.59)	.76
Hispanic	1.84 (0.05-61.84)	.73
White	1 [Reference]	NA
Other race	0.74 (0.45-1.24)	.26
Sex		
Male	1 [Reference]	NA
Female	1.44 (1.12-1.85)	.004
Age, y		
<75	1 [Reference]	NA
≥75	2.46 (1.85-3.26)	<.001
Insurance during treatment		
Medicare	1.15 (0.89-1.49)	.28
Medicaid	1.52 (0.99-2.34)	.06
Commercial health plan	1.12 (0.86-1.46)	.38
Other insurance	0.91 (0.67-1.23)	.53
Uninsured	0.68 (0.49-0.94)	.02
Smoking history		
Never smoker	1 [Reference]	NA
Previous smoking history	1.56 (0.92-2.64)	.10
Practice setting		
Community medical center	1 [Reference]	NA
Academic medical center	2.66 (1.81-3.92)	<.001
Diagnosis year^a^	0.86 (0.80-0.93)	<.001
Bone metastases	0.97 (0.56-1.69)	.92
Brain metastases	2.14 (1.17-3.91)	.01
Anti-infective use prior to treatment	4.04 (2.16-7.56)	<.001
Glucocorticoid use prior to treatment	0.009 (0.007-0.012)	<.001
KRAS positivity	0.88 (0.67-1.16)	.37

Abbreviations: ECOG, Eastern Cooperative Oncology Group; KRAS, Kirsten rat sarcoma virus; ICI, immune checkpoint inhibitor; NA, not applicable; OR, odds ratio; PD-LI, programmed cell death ligand 1.

^a^ Covariate was log-transformed prior to model fitting.

## Discussion

In the PD-L1 high subgroup, chemoimmunotherapy became more commonly prescribed over time. Those with lower performance status were more likely to receive immunotherapy alone given the toxic effects of chemotherapy. Academic clinicians were more likely to forego concurrent chemotherapy for patients with high PD-L1 expression compared with community clinicians, which could reflect a preference for standard treatment guidelines, although more research is needed. Compared with those in the lowest SES quintile, patients in the highest SES quintile were more likely to receive chemoimmunotherapy, which could be explained by well-resourced patients being able to leverage a support system while undergoing chemotherapy. Uninsured patients were more likely to receive chemoimmunotherapy, a trend which should be investigated further. Glucocorticoid use prior to first-line therapy decreased the likelihood of ICI monotherapy. Some literature has hypothesized decreased immunotherapy efficacy in patients receiving glucocorticoids,^6^ potentially leading clinicians to add chemotherapy.

This study has limitations. There were more White patients in this study cohort compared with individuals from minoritized racial and ethnic groups, which may obscure racial disparities. Although we included ECOG status and the presence of bone and brain metastases, we cannot account for markers of rapidly worsening disease, which contribute to the addition of chemotherapy for PD-L1 high patients. Clinicians should consider evidence-based guidelines and the patient’s history to ensure equity when treating aNSCLC.

## Notes

Supplement 1.
eMethods.

eReferences.

Supplement 2.
Data Sharing Statement
