# Supplementary material for: Chemoimmunotherapy vs Immunotherapy Monotherapy Receipt in Advanced Non–Small Cell Lung Cancer
Source: JAMA Netw Open. 2025 Feb 12;8(2):e2459380. doi: 10.1001/jamanetworkopen.2024.59380 (PMC11822537; doi:10.1001/jamanetworkopen.2024.59380)
Supplement: Supplement 1. — eMethods. eReferences. [file jamanetwopen-e2459380-s001.pdf]

Supplemental Online Content

Ahluwalia V, Parikh RB. Chemoimmunotherapy vs immunotherapy monotherapy receipt in advanced non-small-cell lung cancer. *JAMA Netw Open*. 2025;8(2):e2459380. doi:10.1001/jamanetworkopen.2024.59380

eMethods
eReferences

This supplemental material has been provided by the authors to give readers additional information about their work.

© 2025 Ahluwalia V et al. *JAMA Network Open*.

eMethods

The study analyzed patients diagnosed with driver mutation-negative (ALK- and EGFR-negative) aNSCLC from January 2018 to May 2024. The de-identified data originated from approximately 280 cancer clinics (~800 sites of care), with the majority being community oncology settings[5]. Patients were at least 18 years old at advanced diagnosis, had at least two documented clinical visits following diagnosis, and had tumors at stage IIIB, IIIC, or IV at initial diagnosis. Patients who had tumors at earlier stages at initial diagnosis were included if they experienced recurrence or progression after 1 January 2018. Only patients with positive PD-L1 expression (tumor proportion score ≥ 1%) were included. We defined first-line IO monotherapy as nivolumab, pembrolizumab, cemiplimab, atezolizumab, durvalumab, or nivolumab/ipilimumab without another cancer-related therapy. Chemoimmunotherapy was defined as a platinum-based agent with a concurrent IO monotherapy agent.

The cohort was separated into two subgroups: 1) PD-L1 ≥ 50% (PDL1-high) and 2) 1% ≤ PD-L1 < 50% (PDL1-low). A binary logistic regression model was fit for the PDL1-high group with the outcome being receipt of chemoimmunotherapy (encoded as "0") or receipt of IO monotherapy (encoded as "1"). Covariates included in the logistic regression models included socioeconomic status (SES; defined on a five-point scale, with 1 being the "lowest" SES and 5 being the "highest" SES); ECOG status (categorized as 0, 1, or ≥ 2); tumor histology (squamous cell vs. nonsquamous cell histology); self-identified race; ethnicity; gender (male vs. female); age (dichotomized as <75 years old or ≥75 years old); insurance available during treatment (Medicare, Medicaid, commercial health plan, other insurance, or uninsured); smoking history (never smoker vs. previous smoking history); practice setting (academic vs. community); year of advanced diagnosis; presence of bone metastases

© 2025 Ahluwalia V et al. *JAMA Network Open*.

prior to treatment; presence of brain metastases prior to treatment; anti-infective use within the 28 days prior first-line therapy; glucocorticoid use within the 28 days prior to first-line therapy; and KRAS mutational status. We consider KRAS status because KRAS positivity may influence the receipt of chemoimmunotherapy in patients with PD-L1 ≥ 50%[1]. Moreover, we control for the presence of brain metastases and bone metastases to account for measures of disease burden that may influence treatment decisions in this patient population[2,3].

Participant self-identified race and ethnicity were extracted from the database of de-identified electronic health records. Categories for race included Asian, Black, Hispanic, White, and Other Race. Options for ethnicity include Hispanic/Latino or not Hispanic/Latino. Participants were allowed to self-identify race and ethnicity separately. Our data source maps American Indian, Alaska Native, Hawaiian or Pacific Islander, and racial descriptions encompassing multiple racial categories to "Other Race."[4]

Odds ratios larger than 1 indicate a higher likelihood of receiving IO monotherapy whereas odds ratios smaller than 1 indicate a higher likelihood of receiving chemoimmunotherapy.

SES in our cohort is derived from previously-validated standardized measures of socioeconomic status divided into quintiles; this data source has demonstrated that its population is representative of population-level SES in the United States overall[5,6]. Some patients had more than one insurance during treatment (i.e., both commercial health insurance and Medicare). Patients were excluded from analysis if there was no documentation of first-line therapy, the first dose of first-line therapy was documented as prior to the date of advanced diagnosis, or if they tested positive for an *ALK* or *EGFR* mutation. We

© 2025 Ahluwalia V et al. *JAMA Network Open*.

also had access to the mutation status for the following: KRAS, ROS1, BRAF, NTRK1, NTRK2, NTRK3, MET, RET, HER2/ERBB2, and other NTRK mutation. We did not have access to STK11 or KEAP-1 mutation statuses.

The relevant data was extracted into comma-separated-value format using Python 3.11 and statistical analysis was done with Stata version 18.0 (StataCorp). We adhered to the STROBE guidelines when reporting our results. Statistical significance was defined as $p<0.05$.

© 2025 Ahluwalia V et al. *JAMA Network Open*.

eReferences

1. Nakajima EC, Ren Y, Vallejo JJ, et al. Outcomes of first-line immune checkpoint inhibitors with or without chemotherapy according to KRAS mutational status and PD-L1 expression in patients with advanced NSCLC: FDA pooled analysis. *J Clin Oncol*. 2022;40(16_suppl):9001-9001. doi:10.1200/JCO.2022.40.16_suppl.9001

2. Myall NJ, Yu H, Soltys SG, Wakelee HA, Pollom E. Management of brain metastases in lung cancer: evolving roles for radiation and systemic treatment in the era of targeted and immune therapies. *Neuro-Oncol Adv*. 2021;3(Suppl 5):v52-v62. doi:10.1093/noajnl/vdab106

3. Knapp BJ, Devarakonda S, Govindan R. Bone metastases in non-small cell lung cancer: a narrative review. *J Thorac Dis*. 2022;14(5):1696-1712. doi:10.21037/jtd-21-1502

4. Pittell H, Calip GS, Pierre A, et al. Racial and Ethnic Inequities in US Oncology Clinical Trial Participation From 2017 to 2022. *JAMA Netw Open*. 2023;6(7):e2322515. doi:10.1001/jamanetworkopen.2023.22515

5. Guadamuz JS, Wang X, Ryals CA, et al. Socioeconomic status and inequities in treatment initiation and survival among patients with cancer, 2011-2022. *JNCI Cancer Spectr*. 2023;7(5):pkad058. doi:10.1093/jncics/pkad058

6. Yost K, Perkins C, Cohen R, Morris C, Wright W. Socioeconomic status and breast cancer incidence in California for different race/ethnic groups. *Cancer Causes Control*. 2001;12(8):703-711. doi:10.1023/A:1011240019516

© 2025 Ahluwalia V et al. *JAMA Network Open*.
