# Supplementary material for: Chemoimmunotherapy vs Immunotherapy Monotherapy Receipt in Advanced Non–Small Cell Lung Cancer
Source: JAMA Netw Open. 2025 Feb 12;8(2):e2459380. doi: 10.1001/jamanetworkopen.2024.59380 (PMC11822537; doi:10.1001/jamanetworkopen.2024.59380)
Supplement: Supplement 2. — Data Sharing Statement [file jamanetwopen-e2459380-s002.pdf]

Data Sharing Statement

Ahluwalia. Chemoimmunotherapy vs Immunotherapy Monotherapy Receipt in Advanced Non–Small Cell Lung Cancer. *JAMA Netw Open*. Published February 12, 2025.
doi:10.1001/jamanetworkopen.2024.59380

Data
Data available: No
